# SMAD1/5 Signaling in the Early Equine Placenta Regulates Trophoblast Differentiation and Chorionic Gonadotropin Secretion

**DOI:** 10.1210/en.2013-2116

**Published:** 2014-05-21

**Authors:** Victoria Cabrera-Sharp, Jordan E. Read, Stephanie Richardson, Alycia A. Kowalski, Douglas F. Antczak, Judith E. Cartwright, Abir Mukherjee, Amanda M. de Mestre

**Affiliations:** Comparative Biomedical Sciences (V.C-S., J.E.R., S.R., A.A.K., A.M., A.M.d.M.), The Royal Veterinary College, London NW1 0TU, United Kingdom; Baker Institute for Animal Health (D.F.A.), College of Veterinary Medicine, Cornell University, Ithaca, New York 14853; and Biomedical Sciences (J.E.C.), St George's University of London SW17 0RE, London, United Kingdom

## Abstract

TGFβ superfamily proteins, acting via SMAD (Sma- and Mad-related protein)2/3 pathways, regulate placental function; however, the role of SMAD1/5/8 pathway in the placenta is unknown. This study investigated the functional role of bone morphogenetic protein (BMP)4 signaling through SMAD1/5 in terminal differentiation of primary chorionic gonadotropin (CG)-secreting trophoblast. Primary equine trophoblast cells or placental tissues were isolated from day 27–34 equine conceptuses. Detected by microarray, RT-PCR, and quantitative RT-PCR, equine chorionic girdle trophoblast showed increased gene expression of receptors that bind BMP4. *BMP4* mRNA expression was 20- to 60-fold higher in placental tissues adjacent to the chorionic girdle compared with chorionic girdle itself, suggesting BMP4 acts primarily in a paracrine manner on the chorionic girdle. Stimulation of chorionic girdle-trophoblast cells with BMP4 resulted in a dose-dependent and developmental stage-dependent increase in total number and proportion of terminally differentiated binucleate cells. Furthermore, BMP4 treatment induced non-CG-secreting day 31 chorionic girdle trophoblast cells to secrete CG, confirming a specific functional response to BMP4 stimulation. Inhibition of SMAD2/3 signaling combined with BMP4 treatment further enhanced differentiation of trophoblast cells. Phospho-SMAD1/5, but not phospho-SMAD2, expression as determined by Western blotting was tightly regulated during chorionic girdle trophoblast differentiation in vivo, with peak expression of phospho-SMAD1/5 in vivo noted at day 31 corresponding to maximal differentiation response of trophoblast in vitro. Collectively, these experiments demonstrate the involvement of BMP4-dependent pathways in the regulation of equine trophoblast differentiation in vivo and primary trophoblast differentiation in vitro via activation of SMAD1/5 pathway, a previously unreported mechanism of TGFβ signaling in the mammalian placenta.

Placental trophoblast cells perform nutritional, endocrine, and immunomodulatory functions essential to the survival of the developing fetus. Mammalian trophoblast differentiation is tightly regulated through the expression of transcription factors (intrinsic regulators), growth factors (extrinsic regulators), and components of their associated signaling pathways (1). Chorionic gonadotropin (CG) is secreted by both horse and human placenta and is critical to maintenance of early pregnancy. Production of CG is dependent on the differentiation of specialized CG-secreting cells, binucleate (horse) and syncytiotrophoblast (human) (2, 3). These 2 cell types share the expression of the transcription factor glial cells missing 1 (*GCM1*), which is rapidly induced in vivo during differentiation of both equine and human trophoblast (3, 4). The extrinsic factors that regulate terminal differentiation of primary equine binucleate trophoblast cells and CG secretion remain elusive.

The chorionic girdle is a unique transient structure of the equine placenta that gives rise to the endometrial cups. Development of the equine chorionic girdle begins around day 25 of gestation, at which time the uninucleate trophoblast cells undergo a period of rapid proliferation (2). Previous studies of cell morphology (5) and the kinetics of equine (e) CG expression (3, 6), indicate that induction of terminal differentiation of the binucleate trophoblast cells begins around day 31 of pregnancy. The number of binucleate cells then rapidly increases until around day 36–38 (7–10). Several growth factors including epidermal growth factor, vascular endothelial growth factor, hepatocyte growth factor-scatter factor, and TGFβ1 are expressed by the equine endometrium and avascular mesoderm that abut the chorionic girdle (11–16; reviewed by Reference 17), but the functional role of these growth factors in differentiation of chorionic girdle trophoblast is not known.

TGF-β proteins are expressed at the fetal-maternal interface during human pregnancy and are implicated in the promotion of pre- and postimplantation embryonic development (18). Bone morphogenetic protein (BMP)4 is a member of the TGF-β ligand superfamily, which is a large family of dimeric proteins with multifunctional roles including cellular proliferation, differentiation, migration, and apoptosis (19). BMP4 functions through binding to and phosphorylating a type I and II serine/threonine kinase receptor, resulting in the activation of downstream intracellular signals via phosphorylation of the receptor-regulated SMAD (Sma- and Mad-related protein)1/5/8 transcription factors, which complex with SMAD4 to initiate transcription (19, 20). BMP4 signaling can be modified through a number of regulatory inputs, namely availability of the ligand (BMP4), restricted expression of specific type I (BMP receptor, type IA [BMPR1A] and BMP receptor, type IB [BMPR1B]), and type II (BMP receptor type II, BMPR2) receptors, and the presence of the inhibitory pseudoreceptor, BMP and activin membrane-bound inhibitor (BAMBI) (21). TGFβ signaling through SMAD2/3 proteins has been described in a number of studies in normal and preeclamptic placentas (22–29). In contrast, little is known about the function of ligands that signal through SMAD1/5/8 proteins, such as BMP4. Trophoblast lineages have been generated from human embryonic stem cells (ESCs) derived from human blastocysts “primed” by BMP4 supplementation alone or in combination with other factors (30–39). However, the role of BMP4-dependent SMAD1/5 signaling in differentiation of primary trophoblast cells in vitro and in vivo is not known.

There is restricted availability of human placental tissue during early pregnancy and controversy about the availability of in vitro systems that can adequately serve as a model for early events in the human placenta (reviewed by Reference 40), a period in development when embryonic losses are high (41). Studies in the horse may also prove informative to our understanding of the fundamental mechanisms of human trophoblast differentiation (42). CG is a hormone critical to the maintenance of pregnancy that is secreted uniquely by the horse and human placenta. Equine binucleate trophoblast cells and human syncytiotrophoblast cells share expression of growth factors, transcription factors, and components of their associated signaling pathways (3, 4, 42). Further, we have previously shown that it is possible to exploit the late implantation of the equine placenta to isolate pure populations of trophoblast cells at multiple stages of binucleate trophoblast development from the same stallion/mare combination (3). These features of equine pregnancy provide us with a unique resource to dissect molecular events that regulate differentiation of CG-secreting trophoblast cells in vivo, and so integrate molecular data with physiological function.

In this study we investigated the role of BMP-signaling pathways in equine chorionic girdle trophoblast differentiation in vitro and in vivo. Specifically, we elucidate a role for BMP4 in the regulation of terminal differentiation of and subsequent CG secretion from trophoblast cells of the chorionic girdle via activation of the SMAD1/5/8 pathway. Furthermore, we show that when the SMAD 2/3 pathway is inhibited, BMP4-induced terminal differentiation of CG-secreting trophoblast cells is further increased.

## Materials and Methods

### Animals

Mares aged 3–7 years were maintained at the Royal Veterinary College, and animal care was performed in accordance with the Animals (Scientific Procedures) Act 1986 guidelines set by the Home Office and Ethics Committee of the Royal Veterinary College. The reproductive cycle was manipulated, and pregnancies were established as previously described using semen from 3 stallions (3).

### Tissue collection

Conceptuses were recovered by nonsurgical uterine lavage with established methods (3) between days 27 and 34 of pregnancy. Three or four conceptuses per development stage were collected for each individual experiment. The sex of the conceptuses was not determined. Conceptuses were microdissected into chorionic girdle, allantochorion, chorion, yolk sac, bilaminar omphalopleure, and fetus. Human first-trimester placental tissue was obtained as previously described (43) (Wandsworth Local Research Ethics Committee approval reference 01.96.8).

### Culture and stimulation of trophoblast cells

To gain a pure population of trophoblast cells, strips of chorionic girdle were placed into DMEM, and the chorionic girdle trophoblast cells were gently removed from basement membrane and underlying avascular mesodermal cell layer and cultured as per published methods (8). Cells were supplemented with 1–100 ng/mL human recombinant BMP4 (R&D Systems) or an equivalent volume of PBS/BSA. The A83–01 inhibitor, selective for activin receptor type-1B (ACVR1B), TGFβ receptor 1 (TGFBR1) and activin A receptor type IC (ACVR1C) (Tocris), was used at a final concentration of 1 μM. Media were harvested after 72 hours, and supernatant was aliquoted and stored at −20°C.

### Quantification of binucleate cell differentiation and eCG secretion

Binucleate differentiation was quantified by measurement of the number of nuclei per cell using CellTrace BoDIPR TR methyl ester and nuclear stain Hoechst and subsequent fluorescent microscopy after 72 hours in culture. Using a fluorescent microscope, 5 images per well were captured and analyzed using Image J to ascertain the total number of binucleate cells within each image. The concentration of eCG in medium recovered from cultured chorionic girdle cells after 3 days in culture was determined using a pregnant mare serum gonadotropin enzyme linked immunoassay (DRG International), as previously described (44).

### RNA isolation and cDNA synthesis

Total RNA was isolated from snap-frozen equine conceptus tissue, and human placental tissue was stored in RNAlater (Invitrogen), following homogenization by QIAshredder (Qiagen,), using an RNeasy kit (Qiagen) as directed by the manufacturer. RNA (500 ng) was DNase I treated (Invitrogen), and first-strand cDNA synthesis was performed using Moloney murine leukemia virus reverse transcriptase (US Biochemical Corp) as per the manufacturer's guidelines.

### RT-PCR, quantitative RT-PCR, and microarray

PCR was performed using standard methods. Amplification of 15 ng of cDNA was performed in a 20 μL reaction using 10× PCR buffer, 0.2 mM each deoxynucleotide triphosphate, 1.5 mM MgCl (Invitrogen), 0.25 μM each primer, and 1.25 μL recombinant Taq DNA polymerase (Invitrogen). Cycling parameters for PCR were as follows for all amplified cDNAs: an initial denaturation step of 2 minutes at 94°C, followed by 35 cycles of 30 seconds at 94°C, 30 seconds at 59°C, and 1 minute at 72°C, and a final extension step of 10 minutes at 72°C. Ten microliters of each PCR were run on a 1% (wt/vol) agarose gel to visualize PCR products. The PCR products were purified, cloned, and sequenced to confirm the specificity of the PCR product. Primer sequences are shown in Supplemental Table 2.

Real-time RT-PCRs for amplification of equine BMP4, BMPR2, BMPRIA, BAMBI, RGM domain family member B (DRAGON), or the housekeeper gene equine Succinate Dehydrogenase Complex, Subunit A, Flavoprotein (*SDHA*) mRNA were performed using SYBR Green chemistry (KAPA SYBR FAST Universal qPCR kit; KAPA Biosystems). PCR was carried out using a C-1000 thermal cycler and CFX-96 Real time system (Bio-Rad Laboratories) in a total volume of 20 μL. PCRs were carried out for 38 cycles of 30 seconds at 95°C, 30 seconds at 60°C, and 20 seconds at 72°C. A melting curve was set to run from 60°C to 95°C. A dissociation curve was performed after each experiment to confirm that a single product was amplified. For BMP4 and SDHA, a standard curve was generated using known copy numbers of purified PCR products for each gene. Each sample was normalized relative to BMP4 copy number in the chorionic girdle (spatial assay). For BMPRIA, BMPR2, BAMBI, and DRAGON, relative gene expression in days 27–34 chorionic girdle was calculated using the Pfaffl method (45), taking into account the efficiency of the reaction for each individual gene. Primer sequences are shown in Supplemental Table 3. Microarray data used in this study have been previously reported (46).

### Western blot analysis

Tissues were ground and lysed on ice in lysis buffer containing 20 mM Tris-HCl (pH 8), 300 mM KCl, 1% NP-40 (vol/vol), 2.5 mM EDTA, 1 mM VO_4_, 10% glycerol, with a protease inhibitor cocktail (Minicomplete protease inhibitor; Roche). Protein concentrations were determined using Bradford assay (Bio-Rad Laboratories). Protein from mouse spleen (positive control), chorionic girdle, chorion, allantochorion, and yolk sack was diluted with lysis buffer. A total of 40 μg protein per well was loaded and separated by SDS-PAGE on a 10% (wt/vol) polyacrylamide gel before being transferred to a nitrocellulose membrane using a Mini-PROTEAN Tetra cell wet transfer unit (Bio-Rad Laboratories). The membranes were incubated overnight at 4°C in a 1:500 dilution of rabbit antihuman Total SMAD5, phospho-SMAD (pSMAD)1/5, total SMAD2 (Cell Signaling Technology), or pSMAD2 (Millipore Corp) polyclonal antibodies each in Tris-buffered saline-Tween 20 containing 5% (wt/vol) nonfat milk. Sequence alignment confirmed that the human peptide sequences against which the SMAD1/5 and SMAD2 antibodies were directed shared 99%, 100%, and 100% amino acid identities with the corresponding regions of equine SMAD1, SMAD5, and SMAD2, respectively. Membranes were incubated with a 1:10 000 dilution of goat antirabbit IgG secondary antibody conjugated to horseradish peroxidase (Sigma) in Tris-buffered saline-Tween 20 containing 5% (wt/vol) nonfat milk. SMAD proteins were visualized by incubating with ECL plus detection reagents (PerkinElmer) and exposed onto Hyperfilm ECL. To confirm integrity of protein transfer, and as a loading control, membranes were stripped and reprobed for β-actin using a monoclonal mouse β-actin antibody (Sigma) at a dilution of 1:5000. Densitometry analysis of Western blots was carried out using ImageJ 1.47b software.

### Statistics

All statistical tests were performed by using GraphPad Prism 4 statistical software, version 6 (GraphPad). Each experiment was repeated 3–4 times with tissue from a different conceptus on each occasion. Having confirmed the distribution of each data set, the mean (of means) and SEM were calculated for each experimental condition across the 3–4 biological replicates. Quantification of BMP4 expression temporally and SMAD protein expression was compared between the number of days gestation by using one-way ANOVA with repeated-measures and the post hoc Tukey's multiple-comparison test. Quantification of BMP4 expression spatially was compared between tissues and was analyzed by one sample *t* test. BMPRIA, BMPR2, BAMBI, DRAGON, and BMP4 expression in day 27–34 chorionic girdle was compared using Kruskal-Wallis one-way ANOVA. The total number of binucleate trophoblast cells was compared both between the number of days gestation using one-way ANOVA with repeated measures and the post hoc Tukey multiple-comparison test, or by both time and treatment, using repeated measures by 2 factors followed by Tukey or Sidak multiple-comparison test where appropriate. *P* ≤ .05 was accepted as statistically significant in all tests.

## Results

### Receptors for TGFβ superfamily ligands are tightly regulated in the chorionic girdle

Using data generated with a 44 000 equine gene probe expression array (46), relative expression of 7 type I, 3 type II, and 5 accessory TGFβ superfamily receptors in gestational day 34 chorionic girdle tissue was compared to adjacent day 34 chorion tissue (Supplemental Table 1). We searched for receptors that had increased expression in the chorionic girdle (terminally differentiated trophoblasts that secrete CG) compared with chorion (undifferentiated trophoblasts that do not secrete CG). We report that the chorionic girdle preferentially expresses those receptors that are known to bind BMP2, BMP4, and BMP7 (Supplemental Table 1). We found no evidence of up-regulated expression of either TGFβ1-specific type I receptor (TGFβR1) or its type II receptor (TGFBR2); neither did we find evidence for the expression of its SMAD1/5/8-specific pathway type I receptor activin receptor type-II-like I (ACVRL1). TGFβ receptor III (TGFBR3) and endoglin, both associated accessory receptors to TGFβ1, were down-regulated in the chorionic girdle (Supplemental Table 1).

**Figure 1.**
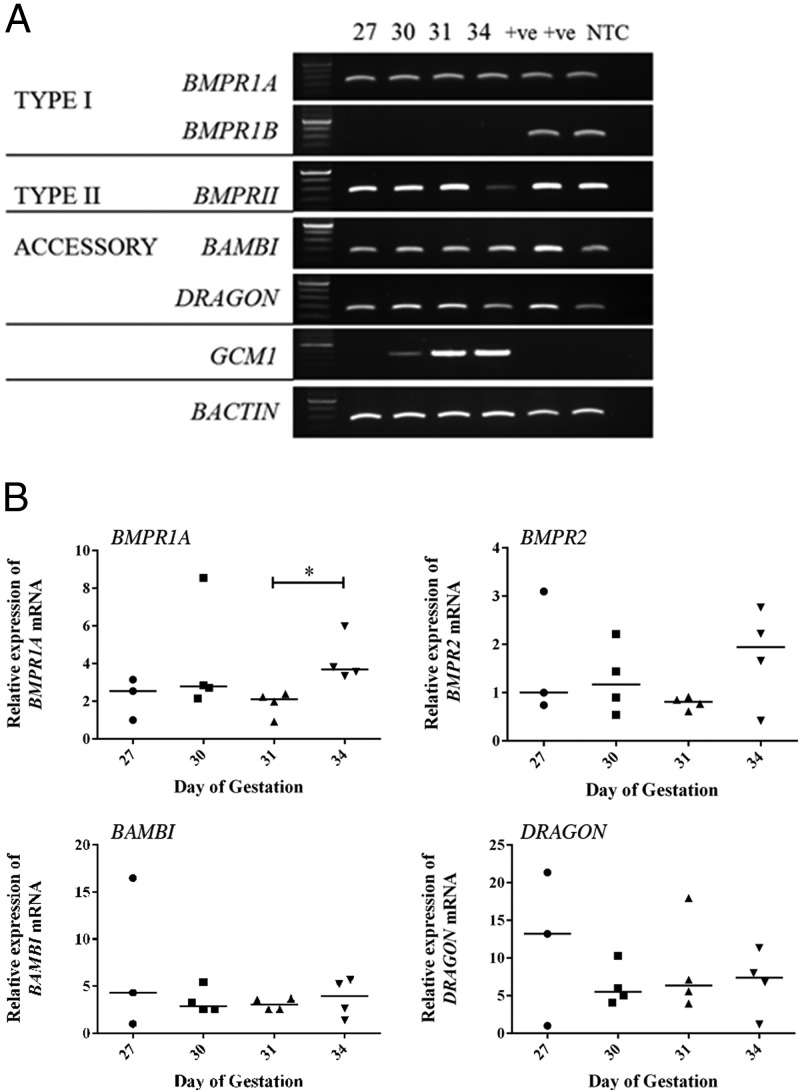
Temporal mRNA expression of type I, type II, and accessory receptors specific for the ligand BMP4 in chorionic girdle tissue from day 27, 30, 31, and 34 conceptuses. A, Qualitative RT-PCR analysis of temporal receptor expression in the chorionic girdle. Amplicons were generated using primers specific for equine *BMPR1A*, *BMPR1B*, *BMPR2*, *BAMBI*, *DRAGON*, *GCM1*, and *BACTIN* (as control) mRNA, and all bands observed in all tissues with each primer set are of the correct predicted size: 306, 343, 364, 264, 269, 450, and 346 bp, respectively. The figure shows typical RT-PCR product gels (n = 3) in which the tissue-specific mRNA expression profile was representative of 3 independent chorionic girdles. NTC, no template control. Equine testis and nonpregnant uterus were used as control tissues (lanes marked +VE). B, Real time quantitative RT-PCR expression of *BMPR1A, BMPR2, DRAGON*, and *BAMBI* mRNA in days 27–34 chorionic girdle. Data points represent the values for individual conceptuses (n = 4, except for day 27 [n = 3] because one sample failed to meet control gene validation parameters and was excluded from the dataset).

Using RT-PCR, we confirmed expression of the type I receptor BMPR1A and type II receptor BMPR2 in chorionic girdle tissue at days 27, 30, 31, and 34 prior to and during the period of binucleate cell differentiation in vivo (Figure 1A). BMPR1B was not detected in any of the chorionic girdle tissues tested. These results suggest that in these chorionic girdle trophoblast cells, BMP may signal through the BMPR1A and BMPR2 dimer. The BMP4-specific accessory receptors, *Dragon* and *BAMBI*, are also expressed at days 27, 30, 31, and 34. As expected, *GCM1* mRNA expression, a marker of chorionic girdle trophoblast cells (3), was confined to the chorionic girdle tissue and was not observed in positive control tissues. Quantitative real time RT-PCR analysis was additionally used to compare temporal expression of these receptors in the chorionic girdle. There was no significant difference in expression levels of BMPR2, BAMBI, and Dragon in the chorionic girdle (Figure 1B) between days 27 and 34 of pregnancy. BMPRIA expression was modestly increased in day 34 chorionic girdle (1.7-fold) when compared with day 31 chorionic girdle (*P* < .05).

### BMP4 stimulates terminal differentiation of chorionic girdle trophoblast cells in vitro

**Figure 2.**
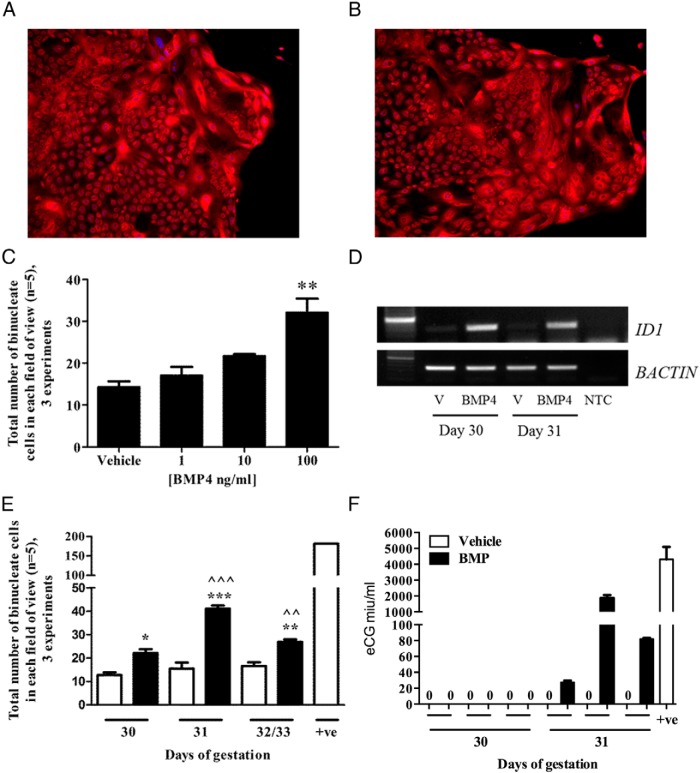
Effects of BMP4 stimulation on terminal differentiation of chorionic girdle trophoblast. Pure populations of chorionic girdle cells from 4 different stages of chorionic girdle development were cultured in the presence or absence of 1–100 ng/mL BMP4, over 72 hours. The total number of binucleated cells was quantified in 5 randomly selected fields for each treatment group; the depicted pooled data represent the mean (of means) + SEM for 3 individual conceptuses at each development stage. Representative images of vehicle treated (A) and 100 ng/mL BMP4 treated (B) chorionic girdle trophoblast cells in culture stained with CellTrace and Hoeschst. C, Quantification of binucleate trophoblast cells following culture in absence or presence of 1, 10, or 100 ng/mL BMP4 for 72 hours. D, DNA-binding protein inhibitor ID-1 (*ID1*) mRNA expression in day 30 and day 31 trophoblast cells in the absence (V) or presence (BMP4) of 100 ng/mL BMP4. E, Quantification of binucleate cells following culture in the presence (black bars) or absence (white bars) of 100 ng/mL BMP4 for 72 hours, derived from 3 different stages of chorionic girdle development on day 34 of pregnancy (positive control). F, Equine CG secretion from chorionic girdle trophoblast cells treated in the absence or presence of 100 ng/mL BMP4 and measured by equine CG ELISA. Data represents mean ± SEM and were analyzed by repeated-measures ANOVA followed by Tukey multiple-comparison test (**, *P* ≤ .01) relative to corresponding vehicle cells, or by repeated measures by 2 factors followed by Tukey or Sidak multiple comparison test (*, *P* ≤ .05; **, *P* ≤ .01; ***, *P* ≤ .001 relative to corresponding vehicle cells. ^^, *P* ≤ .01 (D31 v D32/33); ^^^, *P* ≤ .001 (D30 v D31) relative to corresponding 100 ng/mL BMP4-treated cells.

**Figure 3.**
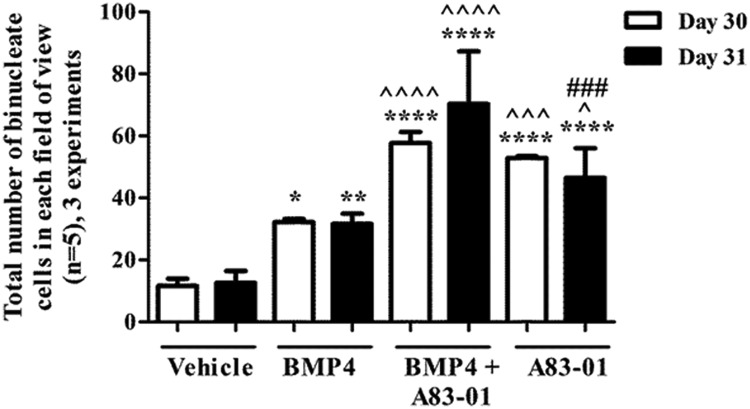
Effect of daily treatment of BMP and SMAD2/3 pathway inhibitor A83–01 on terminal differentiation of chorionic girdle trophoblast cells from day 30 and day 31 conceptuses. Pure populations of chorionic girdle cells were treated daily for 3 days in the presence or absence of 100 ng/mL BMP4, 1 μM A83–01 inhibitor, or both. The total number of binucleated cells was quantified in 5 randomly selected fields for each treatment group; the depicted pooled data represent the mean (of means) + SEM for 3 individual conceptuses at either gestational day 30 or day 31. Data were analyzed by repeated measures by 2 factors followed by Tukey multiple-comparison test (*, *P* ≤ .05; **, *P* ≤ .01; ****, *P* ≤ .0001 relative to corresponding vehicle cells. ^, *P* ≤ .05; ^^^, *P* ≤ .001; ^^^^, *P* ≤ .0001 relative to corresponding 100 ng/mL BMP4-treated cells. ###, *P* ≤ .001 relative to corresponding 100 ng/mL BMP4 plus 1 μM inhibitor-treated cells.

Next, the number of binucleate trophoblast cells within the pure population of chorionic girdle trophoblast cells derived from day 30/31 conceptus' (depicted in Figure 2, A and B) were quantified following culture in the absence or presence of 1, 10, or 100 ng/mL BMP4 for 72 hours (Figure 2C). We observed a significant increase (*P* ≤ .01) in total binuceate cell number when the chorionic girdle trophoblast cells were treated with 100 ng/mL recombinant human BMP4 (Figure 2C). To confirm that recombinant human BMP4 treatment was biologically active on the equine chorionic girdle trophoblast cells, we conducted RT-PCR for DNA-binding protein inhibitor ID-1 (*ID1*), a major transcriptional target of BMP signaling. In comparison with vehicle-treated cells, BMP4 stimulation resulted in increased expression of *ID1* mRNA in chorionic girdle trophoblast cells in vitro (Figure 2D). Subsequently we quantified the total number of binucleate cells following culture in the presence or absence of 100 ng/mL BMP4 for 72 hours, at 3 different stages of chorionic girdle development: day 30, 31, and 32/33 of gestation. Terminally differentiated cells derived from a day 34 conceptus acted as a positive control (Figure 2, E and F). The response to BMP4 treatment was dependent on the development stage. BMP4 treatment significantly increased total binucleate cell number at day 30 (*P* ≤ .05), day 31 (*P* ≤ .001), and day 32/33 (*P* ≤ .01) when compared to corresponding vehicle-treated cells (Figure 2E). Furthermore, the rate of differentiation in day 31 chorionic girdle trophoblast cells was significantly higher than in BMP4-treated cells from day 30 (*P* ≤ .001) and day 32/33 (*P* ≤ .01) chorionic girdle (Figure 2E). Compared with vehicle-treated cells, 100 ng/mL BMP4 induced eCG secretion from immature non-CG-secreting chorionic girdle cells at day 31, but not day 30 (Figure 2F), confirming a specific functional response to BMP4 stimulation. Moreover, daily treatment of 100 ng/mL BMP4 for 3 days can significantly further stimulate differentiation of day 30 cells compared with vehicle-treated cells (*P* ≤ .05), comparable to the differentiation rate seen in day 31 cells (Figure 3). However, daily treatment of BMP4 does not serve to further increase the total binucleate cell numbers in day 31 chorionic girdle trophoblast cells compared with a single dose of 100 ng/mL BMP4 and visualized 72 hours later (Figures 2E and 3).

### Inhibition of SMAD2/3 pathway induces further increases in terminal differentiation of chorionic girdle trophoblast cells in vitro

We report that inhibition of the alternative SMAD2/3 signaling pathway with 1 μM A83–01 (47) in combination with daily treatment of 100 ng/mL BMP4 for 3 days results in a further increased rate of differentiation of chorionic girdle trophoblast in vitro (Figure 3). In both day 30 and day 31 chorionic girdle trophoblast cells a minimum of a 5-fold increase in cell differentiation was observed in cells treated with both BMP4 and A83–01 when compared with the corresponding vehicle-treated cells (*P* ≤ .001, *P* ≤ .001, for both day 30 and day 31). Furthermore, a 2 fold-increase in the number of binucleate cells treated with the combination treatment (BMP4 and A83–01) was observed when compared to the corresponding BMP4 alone-treated cells (*P* ≤ .001, *P* ≤ .001, for both day 30 and day 31). There was no significant difference between the response observed in day 30 and day 31 either with BMP4 alone or when stimulated with BMP4 and A83–01.

The number of terminally differentiated cells in both day 30 and day 31 chorionic girdle trophoblast cells was significantly increased when the cells were treated with A83–01 alone compared with vehicle (*P* ≤ .001) and relative to the corresponding BMP4-treated cells (day 30, *P* ≤ .001; and day 31, *P* ≤ .05) (Figure 3). In addition, there was no significant difference in the number of terminally differentiated cells in A83–01 alone-stimulated day 30 cells compared with the combination BMP4/A83–01 treatment, whereas BMP4 had a significant additive effect in the BMP4/A83–01 combination-treated cells over A83–01 alone-treated day 31 cells (*P* ≤ .001).

### SMAD1/5 signaling is activated during chorionic girdle development in vivo

**Figure 4.**
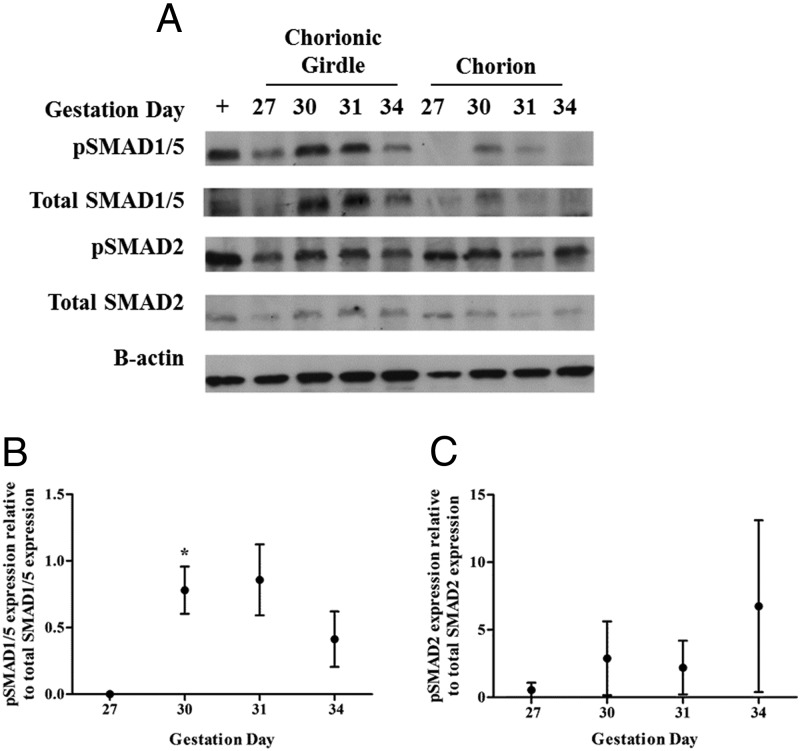
SMAD signaling protein expression in chorionic girdle and chorion tissue isolated from day 27, 30, 31, and 34 conceptuses. Western blots were probed with antibodies raised against human peptide sequences in SMAD1/5 and pSMAD1/5, or SMAD2, pSMAD2 and murine β-actin (as controls) (panel A). Mouse spleen was used as a positive control tissue (+). Each Western blot was quantified by densitometry (panels B and C). Data were analyzed by repeated-measures ANOVA followed by Tukey multiple-comparison test (*, *P* ≤ .05) relative to day 27 chorionic girdle (panels B and C). This representative panel shows the pattern of immunoreactive proteins recognized in equine chorionic girdle tissue protein preparations, confirming that the antibodies directed against the human SMAD1/5 and pSMAD 1/5, SMAD2, and pSMAD2 recognized a single equine protein of approximately 60 kDa. Each panel shows typical Western blots in which the tissue-specific protein expression profile was representative of 3 conceptuses.

Antibodies directed against human phospho-SMAD1/5, human total SMAD1/5, human phospho-SMAD2/3, and human total SMAD2/3 were able to detect a single equine protein of approximately 60 kDa in chorionic girdle of different developmental stages. We subsequently report that there is a significant increase in phospho-SMAD1/5 at day 30, from day 27, indicating that SMAD1/5 signaling is activated during chorionic girdle development in vivo and peaks at day 31, corresponding with the initiation of binucleate cell differentiation (Figure 4, A and B). Furthermore, we observed very little total and phospho-SMAD1/5 in the chorion, indicating that regulation of the SMAD1/5 pathway is specific to the chorionic girdle (Figure 4A). There was no observed regulation of the pSMAD 2/3 proteins in either chorionic girdle or chorion tissue (Figure 4, A and C).

### BMP4 expression indicates the ligand may act on the chorionic girdle primarily through paracrine signaling in vivo

**Figure 5.**
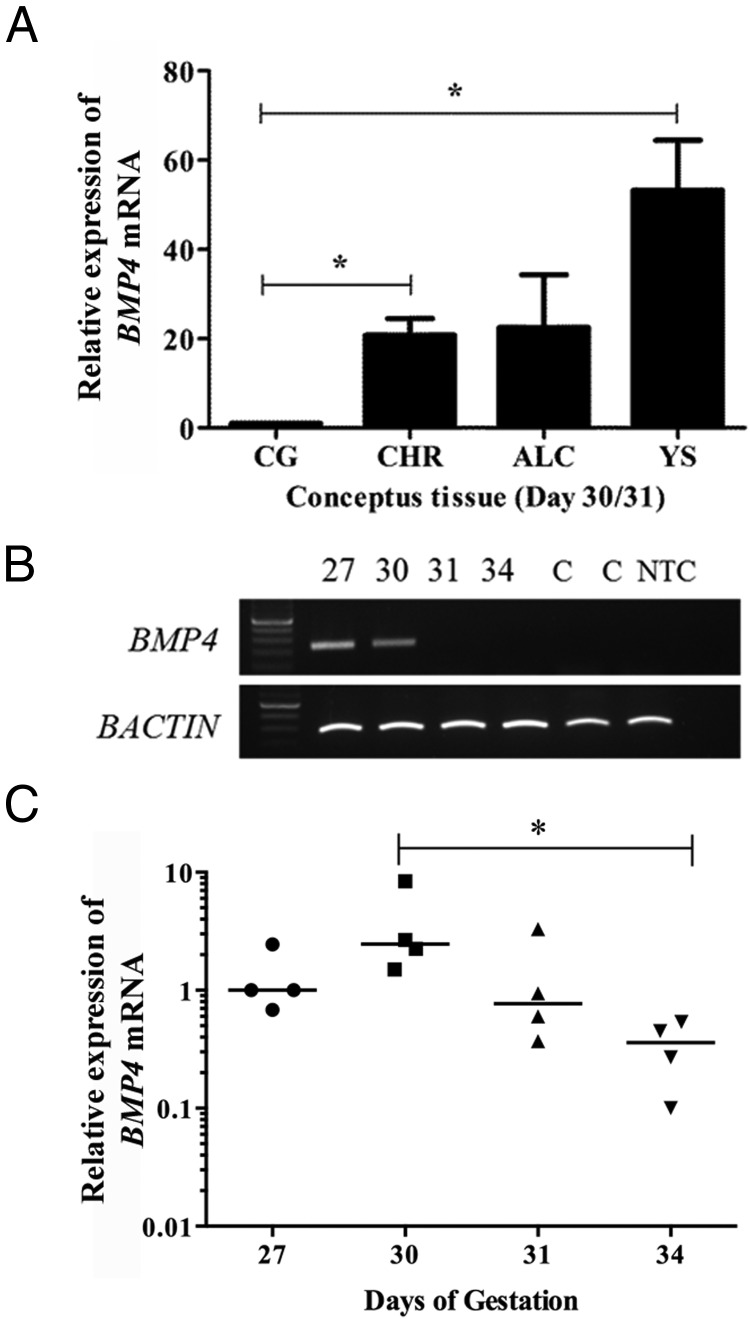
Temporal and spatial expression of *BMP4* mRNA in equine conceptus tissue. A, Quantitative spatial expression of *BMP4* mRNA in chorionic girdle (CG) and adjacent day 30/31 conceptus tissues: chorion (CHR), allantochorion (ALC), and yolk sac (YS). The data represent the median for 3 individual conceptuses. Copy numbers were normalized to 225 000 copies of the housekeeping gene *SDHA*, and data were expressed as fold change over *BMP4* copy number in the chorionic girdle. Data were analyzed by one-way ANOVA with repeated-measures and the post hoc Tukey multiple-comparison test or by one sample *t* test (*, *P* ≤ .05 (0.0136) relative to chorionic girdle), allantochorion relative to chorionic girdle = *P* ≤ .065. B, Qualitative temporal expression of *BMP4* mRNA in chorionic girdle tissue from day 27, 30, 31, and 34 conceptuses. The data represent 3 individual conceptuses. Equine testis and nonpregnant uterus were used as control tissues (lanes marked C). C, Quantitative temporal relative expression of *BMP4* mRNA in chorionic girdle between days 27 and 34. Data represent median values for 4 independent conceptuses for each developmental stage (*, *P* ≤ .05).

Having observed expression of the BMPR1A and BMPR2 receptors in the chorionic girdle, a functional response to BMP4 in vitro, and activation of SMAD1/5 signaling in vivo, we then looked to determine which of the placental membranes expressed the ligand BMP4. In day 30/31 conceptuses, *BMP4* expression in the chorion and yolk sac was 20- to 60-fold higher (*P* ≤ .05) than expression levels in the chorionic girdle, with a trend toward higher expression in the allantochorion (*P* = .065) (Figure 5A). We observed expression of *BMP4* mRNA at days 27 and 30 in the chorionic girdle with no expression detectable by RT-PCR at days 31 and 34 (Figure 5B). Subsequent quantitative RT-PCR analysis showed that *BMP4* mRNA expression was significantly decreased at day 34 (10.9-fold) when compared with day 30 chorionic girdle (*P* < .05) (Figure 5C).

## Discussion

Implantation of the equine placenta occurs relatively late in pregnancy and following differentiation of chorionic girdle trophoblast cells that give rise to the CG-secreting binucleate cells of the endometrial cups. Here we exploited these features of early equine pregnancy to study BMP4 signaling prior to, during, and after terminal differentiation of CG-secreting chorionic girdle trophoblast cells using both in vivo generated trophoblast tissues and ex vivo pure trophoblast cell populations that had experienced minimal manipulation. We found that chorionic girdle cells expressed the receptors for BMP4 in vivo and functionally differentiated in response to BMP4 in vitro. We also demonstrated that SMAD1/5 activity in chorionic girdle in vivo directly correlates with terminal differentiation of chorionic girdle trophoblast with peak SMAD1/5 activity evident at the initiation of chorionic girdle differentiation. A number of reports have demonstrated that BMP4 can drive ESCs down a trophoblast lineage (35–39) across a number of species but the question remains as to whether a requirement for BMP4 is specific to differentiation of ESCs or whether it is a physiologically relevant factor regulating differentiation of committed primary trophoblast in vivo. Here we provide the first evidence of a role for BMP signaling in vitro in primary trophoblast cells and activity of the BMP pathway in vivo at specific periods in early placental development that directly correlate with trophoblast differentiation.

A number of positive and negative regulators of TGFβ signaling through SMAD2/3 have been shown to play a role in regulating trophoblast function (22, 25) and/or are dysregulated in preeclamptic placentas (27, 29). We initially mined a microarray dataset for expression of receptors in the chorionic girdle that can bind the ligand TGFβ1, a TGFβ ligand that is rapidly increased in the endometrium around the time of chorionic girdle development and implantation (2, 13). A comparison of TGFβ receptor expression between chorionic girdle and the adjacent chorion highlighted that the chorionic girdle preferentially expressed the receptors BMPR1A and BMPR2, with little evidence of regulation of receptors that bind TGFβ1. BMPR1A and BMPR2 are genes that encode proteins that form a heterodimeric complex that can bind the ligands BMP2, BMP4, and BMP7 and signal through SMAD1/5/8. These 2 receptors are also expressed in the elongating bovine conceptus (day 17, preimplantation) and the undifferentiated bovine trophoblast cell line, CT1 (48). We have also demonstrated expression of BMPR1A and BMPR2 in first-trimester human chorionic villous tissues (Supplemental Figure 1). The expression of BMPR1A and BMPR2 in the early equine, human, and bovine placenta led us to propose that TGFβ signaling through the alternative pathway, SMAD1/5/8, may also have a conserved and important function in early mammalian placentas.

As far as we are aware, BMP4 expression has not been studied or reported previously in placental or endometrial/decidual tissues. A closely related family member BMP2 has been described in human decidual cells (22). During the period of initiation of differentiation (days 30–31), we found *BMP4* expression levels in the chorion, allantochorion, and yolk sac were between 20- to 60-fold higher than in the chorionic girdle. This suggests that BMP4 is likely to predominantly regulate terminal differentiation of trophoblast in the chorionic girdle through paracrine mechanisms from these adjacent tissues. Following activation of SMAD1/5 in the chorionic girdle at day 30, we observed a decrease in *BMP4* mRNA in the chorionic girdle at days 31 and 34. TGFβ ligand signaling has been shown to also lead to inhibition of expression of the ligand itself, possibly explaining why intrinsic expression of the ligand is lost immediately following the initiation of differentiation.

In these studies, we also provide evidence of BMP signaling in the chorionic girdle in vivo. Phosphorylated SMAD1/5 expression, indicative of BMP signaling, was higher in the chorionic girdle compared with adjacent chorion and peaked at days 30–31 in the chorionic girdle, correlating with the initiation of trophoblast differentiation in vivo (3, 6). This regulation of SMAD1/5 signaling was specific and in contrast to stable activity of the alternative SMAD2 pathway. Indeed, activity of the SMAD2/3 pathway was not specific to the chorionic girdle and was more likely to have a housekeeper role during this phase of trophoblast development in the horse. For example, it may play a role in maintaining proliferation in these tissues during what is a rapid period of placental growth. Phospho-SMAD5 activation decreased again in the chorionic girdle at day 34 of pregnancy, a stage when most of the trophoblast cells have already terminally differentiated. It is plausible that this decrease in SMAD1/5 signaling may be regulated at the level of the receptor, and supporting this possibility we also observed a decrease in the expression of BMPR2 at day 34 when compared with days 27–31.

Studies of the mechanisms that regulate human primary trophoblast differentiation are challenging, primarily due to the limited availability of placental tissue during key windows in early trophoblast differentiation and technical difficulties in separating the trophoblast cells from the surrounding decidual cells (49). Consequently, much attention has been given to developing suitable models of human trophoblast differentiation. One such model is the study of differentiation of human (h) ESCs (49). A number of early reports demonstrated that BMP4 can drive hESCs down a trophoblast lineage (35–39), but this model was brought into question more recently by Bernando et al (50) who state that BMP4 drives hESCs to differentiate primarily toward mesoderm rather than trophoblast. They also found that the methylation state of the BMP-induced cells did not reflect primary trophoblast. Since this conflicting report there has been great effort to further elucidate the role of BMP4 in the hESC model. Amita et al report BMP4 conversion of hESCs to a cell type that expresses a full range of trophoblast markers, has invasive properties and secretes hCG (47). In comparison with hESC induced to differentiate toward trophoblast, equine chorionic girdle trophoblast have lost Eomesodermin (*EOMES*) expression (3), a transcription factor required for trophoblast stem cell renewal (51). Thus the results reported here reflect an observed effect of growth factors on the differentiation of a pure population of fully “committed” trophoblast as they differentiate into subpopulations of trophoblast cells such as those cells that terminally differentiate to secrete CG.

We identified that 100 ng/mL BMP4 alone resulted, after 72 hours, in a doubling of the number of terminally differentiated day 30/31 chorionic girdle trophoblast cells most likely by promoting terminal differentiation because proliferation was not induced with treatment. Response to this treatment was very tightly regulated and developmental stage dependent, with the greatest response to treatment in day 31 cells. The differentiation of these cells was deemed functionally active because we observed that BMP4 induced day 31, but not day 30, cells to secrete eCG compared with vehicle-treated immature nonsecreting day 30/31 cells. Further, BMP4 did not enhance CG secretion from terminally differentiated day 34 trophoblast (Figure 2F, positive control). This supports a role for BMP4 in driving phenotypic change in the trophoblast, therefore leading to the attainment of an altered differentiation state that induces CG expression as opposed to being, specifically, a direct inducer of CG subunit gene expression and/or secretion. In the horse at least, these observations also highlight the very narrow period in development when differentiation events may be initiated and demonstrate the crucial importance of carefully assessing tissue responses at a number of carefully selected windows in early placental development.

Reflecting the mutually opposing interplay that has been reported between SMAD2/3 and SMAD1/5/8 signaling (52, 53), blocking of the SMAD2/3 pathway alone with an inhibitor of the receptors ACVR1B/TGFBR1/ACVR1C resulted in a similar level of differentiation to BMP4 alone. Maximal effect on differentiation was achieved though when BMP4 was combined with the inhibitor of SMAD2/3 signaling. The exact mechanisms that mediate this inhibitory action of SMAD2/3 signaling on BMP signaling in trophoblast is yet to be determined. In mouse mesenchymal cells (C2C12 cell line) (52) and mouse embryonic stem cells (54), TGFβ and Nodal, respectively, can inhibit BMP signaling, activation of SMAD1/5, and induction of BMP-responsive genes via regulation of inhibitory SMAD7. In mouse alveolar epithelial type II cells, TGFβ also has been shown to inhibit BMP signaling but, in this case, acting through antagonism in phosphorylations of the SMADs (53). In the horse, TGFβ1 is rapidly up-regulated in the endometrium between days 30 and 40 of pregnancy, corresponding to the time when the rate of trophoblast differentiation is rapidly decreasing. Future studies that investigate the possible role of TGFβ and Nodal in inhibition of chorionic girdle differentiation are warranted (13).

**Figure 6.**
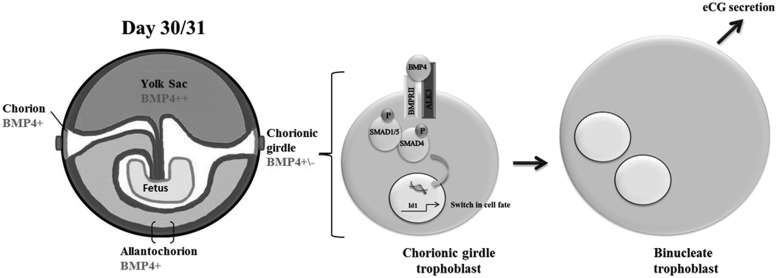
Schematic showing the proposed mechanisms of BMP4 regulated trophoblast differentiation.

In conclusion, our findings support a role for BMP4 signaling in the regulation of terminal differentiation of primary equine trophoblast cells via activation of the SMAD1/5 pathway (Figure 6). The observation of BMP4 signaling in primary trophoblast provides a previously unreported mechanism of TGFβ signaling in the placenta that is likely to be conserved across other mammalian species.
